# Effects of a single interprofessional simulation session on medical and nursing students’ attitudes toward interprofessional learning and professional identity: a questionnaire study

**DOI:** 10.1186/s12909-020-1971-6

**Published:** 2020-03-04

**Authors:** Bryan Burford, Paul Greig, Mike Kelleher, Clair Merriman, Alan Platt, Elize Richards, Neil Davidson, Gill Vance

**Affiliations:** 1 School of Medical Education, Newcastle University, Cookson Building, Newcastle upon Tyne, NE2 1HH UK; 2 Nuffield Department of Clinical Neurosciences, University of Oxford, Oxford, UK; 3 Department of Nursing, Midwifery & Health, Northumbria University, Newcastle upon Tyne, UK; 4 Department of Nursing, Oxford Brooks University, Oxford, UK; 5 Medical School, University of Oxford, Oxford, UK

**Keywords:** Simulation, Professional identity, Social identity theory, Interprofessional learning, Interprofessional simulation, RIPLS, Medical students, Nursing students

## Abstract

**Background:**

Participation in simulation-based interprofessional education (sim-IPE) may affect students’ attitudes towards interprofessional learning (through gaining experience with others) and their professional identity (by increasing the ‘fit’ of group membership). We examined this in two questionnaire studies involving students from four universities in two areas of the UK.

**Method:**

Questionnaire data were collected before and after students took part in a sim-IPE session consisting of three acute scenarios. Questionnaires included the Readiness for Interprofessional Learning Scale (RIPLS) and measures of professional identity derived from the social identity theory literature. In Study 1, only identification with Professional Group (doctor or nurse) was measured, while in Study 2 identification with Student Group (medical or nursing student) and the immediate interprofessional Team worked with in the simulation were also measured.

Linear mixed effects regression analysis examined the effect of the simulation session, and differences between medical and nursing students, sites and identity measures.

**Results:**

A total of 194 medical and 266 nursing students completed questionnaires.

A five-item subset of RIPLS (RIPLS_Core_) was used in analysis. In both studies RIPLS_Core_ increased for all groups following participation in sim-IPE, although this was larger for nursing students in Study 1. Nursing students had consistently higher RIPLS_Core_ scores than medical students at one site.

Effects of the session on identity varied between sites, and dimensions of identity. Notably, while positive emotions associated with group membership (Ingroup Affect) increased for Student Group, Professional Group and Team, the sense of belonging (Ingroup Ties) and importance (Centrality) of the group increased only for Team. Nursing students had consistently higher identification scores than medical students.

**Conclusions:**

Participation in a sim-IPE session can improve attitudes towards interprofessional learning. It can also enhance professional identity, particularly as related to emotional aspects of group membership, with possible benefits for wellbeing. Changes in identification with the immediate Team suggest positive psychological consequences of ad hoc Team formation in the workplace.

Differences between medical and nursing students suggest their differing opportunities to work with other professions during training may change baseline attitudes and identity. However, a single sim-IPE session can still have an additive effect.

## Background

Simulation-enhanced interprofessional education (sim-IPE [1], also known as interprofessional simulation-based education [IPSE]) is the delivery of interprofessional learning (IPL), involving members of different professional groups, in a simulated work setting. At an undergraduate level, sim-IPE can mitigate against limited opportunities students may have to work together in clinical placements, and provide a safe environment in which to take on the role of independent practitioner.

There has been widespread interest in sim-IPE, indicated by several recent reviews [2–7]. However, little of this work has included medical and nursing students – surprisingly, considering the extent to which doctors and nurses work together in practice. Reviews also note methodological weakness, with many studies conducted in single sites with small sample sizes and limited outcome measures.

We describe two studies conducted with medical and nursing students from four universities. These considered the impact of sim-IPE on participants’ attitudes towards IPL and professional identity. We are not aware of any previous work which has considered these constructs together in the context of simulation.

Attitudes to IPL provide an indication of how engaged people may be with such activity. A number of measures of these attitudes have been described, but the most common in the literature is the Readiness for Interprofessional Learning Scale (RIPLS [8]). While RIPLS has been found to be sensitive to participation in interprofessional simulation [9], high quality studies examining such effects appear to be scarce.

Professional identity meanwhile is increasingly recognised as an important element of professional practice [10] and may have consequences for medical students’ wellbeing [11, 12]. Students can identify as professionals from as early as their first year of undergraduate training [13, 14], but the process of psychologically ‘becoming’ a professional is particularly important as students approach qualification. Alongside this, enhancing identity as a team collaborator, rather than a member of a single profession, can improve interprofessional practice in the workplace [15]. Despite the importance of this concept, we found no studies looking at the direct effects of participating in simulation on professional identity, although there is evidence that sim-IPE can challenge stereotypes held about professional groups [16, 17].

As considered through the lens of social identity theory [18], professional identity may be defined as an individual’s self-categorisation as a member of their professional group – a doctor or a nurse. The relative dominance, or salience, of a particular group membership depends on two cognitively-based elements: the ‘readiness’ of an individual to adopt it, and its ‘fit’ [19]. Readiness is determined by context, meaning that different potential identities are accessible in different settings. For healthcare students in a sim-IPE context, potential identities include their current student group and their eventual professional group, but may also include the immediate interprofessional team. Fit may be determined by comparing oneself against perceived attributes of that group (‘normative fit’), which may include external criteria such as being qualified, but also individual knowledge, skills and behaviour. We suggest that acting in the role of a qualified professional in a simulation will increase the normative fit of that professional group.

### Differences between medical and nursing students

There is evidence that attitudes to IPL, and professional identity, differ between professional groups. Studies have found that nursing students score more highly than medical students on the RIPLS [13, 20, 21], but there have been mixed findings with regard to professional identity [13, 22]. These differences may reflect differences in educational experience. Nursing students’ undergraduate training generally involves working as active members of clinical teams from the outset, compared to relatively short, and more peripheral, placements undertaken only by senior medical students. Baseline measures may therefore be higher, and effects of simulation less, for nursing students than for medical students.

### The current studies

We describe findings from two questionnaire studies. Study 1 was based at a single location (Newcastle upon Tyne, UK), while Study 2 included a second (Oxford, UK). Both studies examined two central hypotheses:

1. Participation in the session will be associated with improved attitudes to IPL and higher identity measures.
2. Nursing students will initially have more positive attitudes to IPL, and higher professional identity, than medical students.

Study 2 examined additional hypotheses:

3. Effects will differ between sites with different programmes, and where medical students have or have not already completed final examinations.
4. Identity measures reflecting identification with Professional Group (doctor or nurse), Student Group (medical or nursing student) and the immediate interprofessional Team will differ.

These studies add to the literature in a number of ways which enhance our understanding of how simulation affects students. Attitudes towards interprofessional learning and different aspects of identity have not been considered together in this way before. The nature of the IPE is also different, in that the studies consider the impact of a single interprofessional session, rather than the longer IPE interventions described in the literature. Finally, the interprofessional teams involved are truly ad hoc, identifying the impact of a team which has not had the opportunity to become acquainted, again in contrast to earlier studies [16].

## Methods

Both studies used a pre-post design to explore changes in measures following participation in a sim-IPE session, conducted as part of routine teaching.

### Participants, setting and educational context

Simulation sessions in Newcastle and Oxford were broadly similar, except where noted. Sessions took place in purpose-built facilities with high-fidelity patient simulators and genuine clinical equipment representing an acute bay in a ward setting. Each session was attended by up to nine medical students and up to six nursing students.

Medical students were in clinical placement blocks in their final year, and 3–4 months from starting work as doctors. However, while Newcastle medical students were still 2 months from their final examinations, Oxford medical students had completed finals and knew their results. Most nursing students were in their second year, although some were recruited from years 1 and 3. In Newcastle, participation in the simulation sessions was compulsory for medical students, and voluntary for nursing students. In Oxford the situation was the reverse. All participants were notified of the voluntary nature of the research in advance.

Each session comprised three acute care scenarios in which students could practice ‘ABCDE’ (airway, breathing, circulation, disability, exposure) assessment – examples included sepsis, anaphylaxis and acute abdominal pain. Students were not informed of the possible scenarios in advance. Scenarios were designed to reflect best practice in clinical simulation [23].

In each scenario, initial assessment was conducted by nursing students. They called medical students in the role of junior doctors, who then carried out their own assessment and began management before the patient deteriorated. This phase of the scenarios involved extensive communication between the medical and nursing students. In Newcastle, scenarios terminated when the medical students called a senior for help, while in Oxford scenarios could continue beyond this point into resuscitation, or even manikin ‘death’. Each scenario took 20–40 min to unfold, followed by a 30–40 min debrief with teaching faculty.

In Newcastle, medical students entered the scenario in pairs, and in Oxford in threes. In Newcastle one student was designated as ‘lead’ in advance (that is, not manipulated as part of the research study), meaning they took responsibility for assessment and management of the patient and the decision to call for senior help, while in Oxford a lead was not nominated by faculty but could be agreed among students, or emerge during the scenario. The remainder of the groups observed the scenario remotely through a video link.

A member of simulation faculty was also present in the simulation room, providing details of observations that were not available through the patient simulator (eg capillary refill time). In some sessions, a clinical educator was also present in the observation room providing commentary and facilitating discussion. Authors AP, MK and ND in Newcastle, and PG, ER and CM in Oxford were involved in the design and delivery of sessions.

### Procedure

Following standard briefing from teaching faculty, a researcher introduced the study, and invited students to complete the pre-session questionnaire. The simulation session then proceeded as normal. Following the final scenario and debrief, the researcher asked all participants to complete the second questionnaire. The post-session questionnaire was administered at this point for logistical reasons so as not to intrude on the educational delivery of the session, but, as debriefing is an integral part of simulation-based education, this also provides ecological validity.

### Questionnaire materials

Questionnaires were anonymous, with pre- and post-session forms linked using unique reference numbers. In addition to scale items described below, the pre-session questionnaire asked for participants’ age, gender and previous experience of simulation. The post-session questionnaire also asked which role students had taken in the session (lead, other participant or remote observer).

#### Attitudes towards interprofessional learning

Questionnaires in both studies used the 19-item RIPLS measure [8], with a five-point response scale from ‘strongly disagree’ to ‘strongly agree’. Bearing criticisms of RIPLS in mind [24], in analysis we used a uni-dimensional measurement based on an item response theory analysis [25] published since our data collection. This measure was derived from the mean of five items identified as the most informative in that analysis. These five ‘RIPLS_Core_’ items are (with their numbering and associated subscales from the original publication of RIPLS [8]):

- Item 3. Shared learning with other health care students will increase my ability to understand clinical problems. (Teamwork and collaboration).
- Item 4. Learning with health care students before qualification would improve relationships after qualification. (Teamwork and collaboration).
- Item 8. Team-working skills are essential for all health care students to learn. (Teamwork and collaboration).
- Item 11. It is not necessary for undergraduate health care students to learn together. (Professional identity, reverse-scored).
- Item 15. Shared learning will help to clarify the nature of patient problems. (Professional identity).

All scale items are included in Additional file 1.

#### Professional identity

In Study 1 we followed earlier work in medical education [13, 14] by using a measure of identity derived from social identity theory, and extensively used in organisational settings [26]. This includes 10 items reflecting different dimensions of identification – awareness, evaluation and affect – but is treated as a single measure. We refer to this simply as *Strength* of identification. We also used a 4-item scale assessing the *Importance* of the group to the individual [27].

In Study 2 we sought further refinement of the identity measure by using a scale with three explicit subscales reflecting different dimensions of identity, again derived from social identity theory [28]. *Centrality* reflects a group’s ‘enduring psychological salience’ ([28], p., 253) for an individual, linked to their readiness to adopt an identity. It is analogous to the ‘importance’ scale in Study 1. *Ingroup Affect* reflects positive feelings associated with the group, while *Ingroup Ties* reflects the interpersonal experience of group membership and a sense of ‘belonging’. Both Ingroup Affect and Ingroup Ties have elements of the Strength scale in Study 1, although Cameron demonstrated that it was most strongly statistically associated with Ingroup Affect [28]. Items in Study 2 relating to Professional Group were adapted to the future tense, eg ‘In general, the fact that I am going to be a doctor is an important part of my self-image’.

Study 1 considered participants’ identification only with their eventual professional group (ie doctor or nurse). Study 2 also considered student group (medical or nursing student) and the interprofessional team in the simulation scenario. In analysis we refer to these groups as the ‘Target’ of the identity measures.

### Analysis

To evaluate internal consistency, Cronbach’s alpha was calculated for all scales. Sample sizes were too small to consider scale dimensionality, and so scale structures established in the literature were used.

#### Missing data

Respondent-mean substitution [29] was used to generate scale scores if just one item had been omitted. In Study 1, 10 missing values from 8 respondents, and in Study 2, 36 values from 25 respondents, were generated in this way. If more than one item was omitted, no scale score was calculated (this applied to two respondents in Study 1, and 25 in Study 2, many of whom did not complete all of the second questionnaire due to time constraints or printing error).

#### Regression modelling

The main analysis used linear mixed effects modelling, a form of linear regression suitable for repeated measures designs, which allows analysis of unbalanced datasets [30]. Analysis used the lme4 package in R [31, 32].

RIPLS_Core_ and the identity subscales were used as outcome variables in separate analyses. We used a criterion-based approach to model selection to identify whether hypothesised effects contributed to these scores. Starting with a model including all hypothesised effects, the contribution of each was tested using the drop1() function in lmer4 [31]. Final models retained only predictors whose removal would significantly reduce model fit.

An a priori comparison tested whether those Newcastle medical students designated as ‘lead’, with a nominally more active role in the simulation, would exhibit greater changes in measures than other participants. No such effects were found, and so role was not included in models. Initial model building also found that previous experience of interprofessional simulation did not contribute to any models.

In all regression models, respondent was included as a random intercept to control for individual differences in responses, while other predictors were included as fixed effects. Factors included in Study 1 and retained as significant effects in at least one final model were:

- *Pre-Post* (to identify changes in measures following the simulation session).
- *Participant Group* (to identify differences between nursing and medical students).

Analyses for Study 2 included these and additional effects:

- *Site* (to identify differences between Oxford and Newcastle).
- *Target* of identity measure (to identify differences between identity measures referring to Professional Group, Student Group and Team).

Two- and three-way interactions were included in initial models to examine whether effects were consistent across levels of the other factors.

Follow-up analyses on final models used the emmeans package [33] to calculate and compare estimated marginal means (the means derived from the model, rather than the sample data). These are reported in place of regression coefficients to aid clarity of interpretation (coefficients are provided in Additional file 2 along with all estimated marginal means). All *p*-values for multiple comparisons were adjusted using the Tukey HSD method.

## Results

### Respondents

There were 126 participants in Study 1 (88 medical and 38 nursing students), and 186 across both sites in Study 2 (106 medical and 80 nursing students). Demographics are summarised in Table 1.

**Table 1:** Sample demographics

	Study 1 (*n* = 126)		Study 2 (*n* = 186)			
	Newcastle only		Newcastle		Oxford	
	Medicine (*n* = 88)	Nursing (*n* = 38)	Medicine (*n* = 50)	Nursing (*n* = 23)	Medicine (*n* = 56)	Nursing (*n* = 57)
Sex	37 female, 49 male, 2 unknown	36 female, 2 male	25 female, 25 male	23 female, 0 male	31 female, 25 male	50 female, 6 male, 1 unknown
Mean age (years)	24.7	23.7	24.0	26.5	24.4	24.6

In Study 1, few participants had experience of interprofessional simulation, with just 18% of nursing students (*n* = 7) and 35% of medical students (*n* = 31) reporting any such experience. In Study 2 a majority of medical students at both sites (n = 29, 58% at Newcastle, *n* = 29, 52% at Oxford), but still a minority of nursing students (*n* = 8, 35% at Newcastle, n = 8, 14% at Oxford), had some experience. The Newcastle MBBS programme is distributed across a number of sites in the clinical years 3 and 5, and the change in sim-IPE experience between the Newcastle cohorts could be down to a difference in earlier experience in Year 3. However, we do not have data to demonstrate this and it may simply arise from random variation in experience.

#### Internal consistency of measures

The internal consistency of measures was generally adequate (see Table 2). For the pre-measures of RIPLS_Core_ in Study 1, and Centrality of student group in Study 2, it was marginally below the conventional threshold of alpha> = 0.7, but acceptable for the short 4 and 5 item scales used.

**Table 2:** Cronbach’s Alpha for subscales

	Study 1		Study 2	
	Pre	Post	Pre	Post
RIPLS_Core_	0.62	0.81	0.72	0.80
Identity Strength	0.88	0.91		
Identity Importance	0.72	0.76		
Ingroup Ties: Professional			0.79	0.80
Ingroup Ties: Student			0.85	0.79
Ingroup Ties: Team			0.76	0.80
Centrality: Professional			0.73	0.76
Centrality: Student			0.68	0.74
Centrality: Team			0.74	0.85
Ingroup Affect: Professional			0.86	0.87
Ingroup Affect: Student			0.85	0.86
Ingroup Affect: Team			0.80	0.84

### Mixed effects regression: effects of simulation session and participant group

Our central hypotheses concerned the effects of the simulation session (the Pre-Post factor) and being a medical or nursing student (Participant Group). We expected an increase in all measures, and nursing students to score more highly than medical students. In both studies we observed both of these effects, but significant interactions showed that they were not consistent.

#### Effects on RIPLS_Core_

The RIPLS_Core_ measure increased for all groups across both studies. In Study 1, the difference was smaller for medical than nursing students, indicated by a significant Pre-Post x Participant Group interaction, but significant for both (estimated marginal means [em-means] are given in Table 3). In Study 2, the increase over a session was significant, with no interactions, indicating it was similar for medical and nursing students, and Newcastle and Oxford (overall em-mean pre = 4.38, se = 0.03; em-mean post = 4.60, se = 0.04).

**Table 3:** Estimated marginal means for Pre-Post x Participant Group interaction: RIPLS_Core_ (Study 1 – Newcastle only)

	Estimated marginal means (se)		
	Pre	Post	t-ratio
Medicine	4.20 (0.05)	4.33 (0.05)	2.937*
Nursing	4.52 (0.07)	4.88 (0.07)	5.501****
t-ratio	3.708**	6.377****	

*p*-values adjusted by Tukey method for comparing a family of 4 estimates. *p < 0.05, **p < 0.01, ****p < 0.0001

At Newcastle, medical students had lower RIPLS_Core_ scores than nursing students, as expected, but this was not true at Oxford (see Table 4). Newcastle medical students scored lower than their Oxford peers, while the two nursing groups did not differ.

**Table 4:** Estimate marginal means for Participant Group x Site interaction: RIPLS_Core_ (Study 2 – Newcastle and Oxford)

	Estimated marginal mean (se)		
	Medicine	Nursing	t-ratio
Newcastle	4.19 (0.06)	4.73 (0.08)	−5.344****
Oxford	4.43 (0.05)	4.60 (0.05)	ns
t-ratio	3.170**	ns	

*p*-values adjusted by Tukey method for comparing a family of 4 estimates.

**p < 0.01, ****p < 0.0001

Overall, the hypothesised effect of the simulation session was observed on RIPLS_Core_ scores. The hypothesised difference between medical and nursing students was partly supported, but the pattern at Oxford suggests this is not just a difference between professions.

#### Effects on identity scales

Hypothesised effects were observed on the different identity measures in the two studies. Results suggest that measures of identity are affected by participation in simulation, but that effects vary with specific aspects of identity, and sites.

The effect of Participant Group was consistent across all measures, with nursing students scoring higher than medical students, even within interactions. The Importance scale in Study 1 showed this with no interactions (nursing student overall em-mean = 5.50, se = 0.15; medical student overall em-mean = 4.57, se = 0.10).

Pre-post differences were more variable. In Study 1, there was no change for nursing or medical students on the Importance scale, and only nursing students’ scores on the Strength scale increased (see Table 5).

**Table 5:** Estimated marginal means for Pre-Post x Participant Group interaction: Identity Strength (Study 1 – Newcastle only)

	Estimated marginal means (se)		
	Pre	Post	t-ratio
Medicine	5.50 (0.08)	5.60 (0.08)	ns
Nursing	6.11 (0.12)	6.39 (0.12)	4.138***
t-ratio	4.446****	5.693****	

*p*-values adjusted by Tukey method for comparing a family of 4 estimates

***p < 0.001, ****p < 0.0001

In Study 2, some significant changes were found for all subscales. Ingroup Affect – the positive feelings associated with group membership – improved consistently, with no interaction with Participant Group, Site or Target (Pre overall em-mean = 4.30, se = 0.04; Post overall em-mean = 4.38, se = 0.04).

**Fig. 1:**
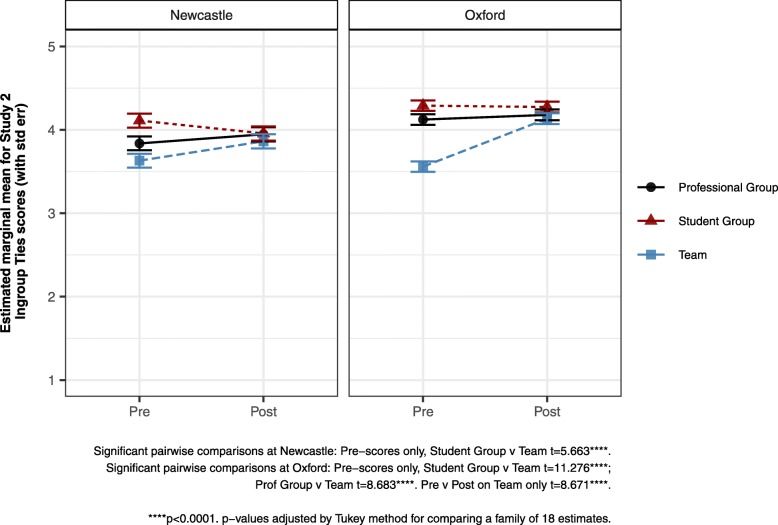
Pre-Post x Target x Site interaction: Ingroup Ties (Study 2 – Newcastle and Oxford)

By contrast, Ingroup Ties and Centrality for Professional and Student Group did not change. These measures did change for Team however – Centrality across both sites (Table 6), Ingroup Ties just at Oxford (Fig. 1). The Centrality result is therefore comparable with that for Importance in Study 1, where only Professional Group was addressed.

**Table 6:** Estimated marginal means for Pre-Post x Target interaction: Centrality (Study 2 – Newcastle and Oxford)

	Estimated marginal means (se)		t-ratio
	Pre	Post	
Professional	4.01 (0.05)	4.01 (0.05)	ns
Student	3.87 (0.06)	3.75 (0.06)	ns
Team	3.36 (0.05)	3.55 (0.06)	−3.373*
t-ratio	Prof-Student: ns Prof-Team: 11.645**** Student-Team: 8.902****	Prof-Student: 4.419*** Prof-Team: 8.117**** Student-team: 3.479*	

*p*-values adjusted by Tukey method for comparing a family of 9 estimates

*p < 0.05, ***p < 0.001, ****p < 0.0001

**Fig. 2:**
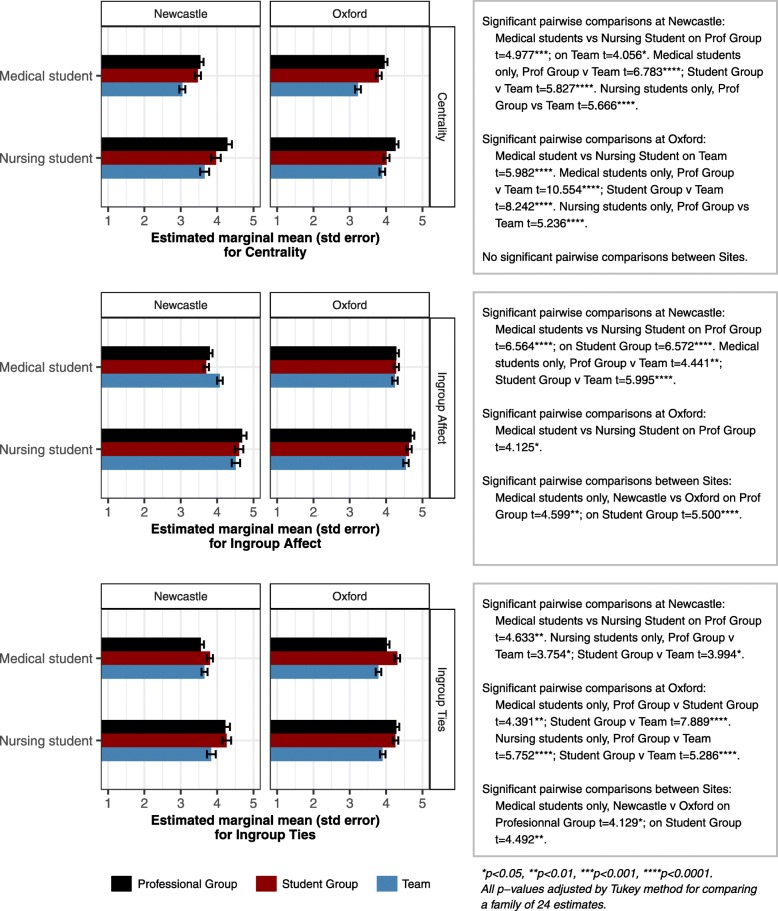
Target x Participant Group x Site interaction for identity variables (Study 2 – Newcastle and Oxford)

All three scales showed an interaction between Participant Group, Target and Site (ie collapsing Pre and Post measures, see Fig. 2). This can be hard to interpret, as there are a number of differences in the patterns of significant pairwise contrasts between variables and sites. However, a common element is that it appears that Newcastle medical students’ scores are lower than their Oxford counterparts, and Newcastle nursing students. That said, their score for Ingroup Affect for Team is *relatively* higher when compared to Professional Group and Team: while Newcastle medical students indicate less strong identification with any group, their identification with the immediate Team is stronger than with Profession Group or Student Group.

## Discussion

Two studies found support for three hypothesised effects – of participation in a single sim-IPE session, of being a medical or nursing student, and of site – on nursing and medical students’ attitudes to interprofessional learning, and professional identity.

### Effects on attitudes towards interprofessional learning

In both studies, both medical and nursing students’ attitudes towards interprofessional learning were more positive following the session, albeit from a high baseline. Intuitively it seems that ‘working’ together in an educational setting may illuminate the contribution of the other group, and so make further interactions more appealing.

Nursing students had higher RIPLS_Core_ scores than medical students at Newcastle, echoing earlier findings [13, 20], but this difference was *not* significant at Oxford. The hypothesised difference was based on nursing students’ having had more regular contact with a range of other health care practitioners, and we can surmise there may be an unidentified difference between medical students’ experience. However, we do not know if this is due to differences in curricula, opportunistic experience, delivery of scenarios, or linked to the medical students being post-finals.

### Differences in identity measures

Effects varied between measures of different dimensions of identity, and measures referring to Student, Professional and Team identity in Study 2. Effects on Student and Professional identity were similar. However, use of the future tense for the latter may have confounded this, with ‘being a student’ effectively synonymous with ‘going to be a professional’ in terms of self-categorisation. Some effects on Team were different, and we consider those separately below.

#### Professional and student identity

Results from Newcastle supported the hypothesis – based on the inference from social identity theory that the greater experiential learning of nurses will provide greater fit – that nursing students would have higher identification than medical students. The contrasting pattern in Oxford, where medical and nursing student identity were more similar, may however also be explained by normative fit, as these medical students had already passed finals, passing a formal threshold towards *objectively* being doctors. However, as noted above there may also be other differences in learning experience between Newcastle and Oxford medical students.

Changes in identity scores varied between the measures. In Study 1, nursing students’ scores increased on the Strength scale, while medical students’ did not. The comparable scales from Study 2 showed contrasting results. Ingroup Affect, the positive emotions associated with group membership, increased for all groups, while Ingroup Ties, the sense of belonging, increased for none. It is possible therefore that medical and nursing students in Study 1 were responding to the Strength scale – which contained elements of affect and belonging – in different ways.

Ingroup Affect has been associated with self-esteem derived from group membership [28], and our findings suggests that participants felt more positively about being students, professionals and team members after the simulation. By contrast, the sense of belonging indicated by Ingroup Ties did not change. This subscale most represents the concept of *fit* described in the social identity approach, and so this finding does not support our hypothesis. However, while it contains items reflecting similarity, it is conceptualised as an *affective* bond which may not be sensitive to increased experience in a role.

No changes for either group were observed in the Importance (Study 1) and Centrality (Study 2) scales. These reflect a stable cognition, linked to the concept of ‘perceiver readiness’ [19], and as such, a lack of change is understandable. Pre- and post- responses were given in the same context, and so all groups would be equally salient.

Measures were all high before the simulation, reflecting identities which are already psychologically established. While there is no evidence of a ceiling effect, there is therefore a smaller potential for change.

### The immediate team

Team-referent measures showed a distinct pattern of change between pre- and post- measures. As well as the change in Ingroup Affect also observed for Student and Professional measures, Team Centrality increased for medical and nursing students at both sites, while Ingroup Ties increased at Oxford.

This suggests some validation of our measures. Team was a new group membership, and so lower salience than established Professional and Student groups is to be expected. The fact that pre- scores for Team are relatively high is potentially more surprising, but may be evidence of the ‘minimal group effect’ described in the social identity theory literature – where simply being placed in a group is sufficient to elicit a sense of membership [34].

The finding that an ad hoc interprofessional team can be subjectively meaningful to students is important because healthcare is often delivered by such teams, and team identity has been found to affect interprofessional performance more than professional identity [15]. A team may constitute a ‘common ingroup identity’, a group membership shared by different ingroups which has the potential to reduce ingroup conflict [35]. Our results do not directly allow conclusions on this, but the existence of an ad hoc, cross-professional, identity gives some credence to its existence, if not impact.

### Effect of observation

We did not identify a difference between those medical students who were designated an active role in the simulation and those observing. This resonates with previous research showing that participants and observers demonstrate similar learning outcomes [36, 37]. While one study suggests that observers are less emotionally involved with simulation [38], our findings suggest the psychological impact in terms of identity and attitude is similar for observers and participants.

### Limitations

The study was limited to just two locations, and while we have found that effects vary between sites, we can only speculate as to the effects of specific curricula elements.

Controls were limited because data were collected as part of a live educational intervention. There was no control group exposed to a uniprofessional simulation, meaning we cannot exclude the possibility that the observed effects would also be found in a uniprofessional session. In Newcastle, medical students had designated roles, but in Oxford, and among all nursing students, any differences in roles were emergent and not recorded. These and other unrecorded variations in scenarios, may have confounded observed effects. Nonetheless, our studies have ecological validity, deriving from their real educational setting.

There is a possible effect of the simulation sessions being voluntary for some participants, and compulsory for others. Across variables there were trends that student groups who volunteered to take part in the simulation (nursing students in Newcastle, medical students in Oxford) gave higher scores than those for whom participation was compulsory. This was outside the control of our research, but it is possible that a self-selection bias for participation in the educational intervention itself was apparent in our research data.

The absolute differences observed in scales are small, but there are no clear criteria to evaluate the practical implications of these differences, and they are in line with those reported in the literature and interpreted as meaningful [13]. These are measures of attitudes rather than practice, and our focus is on identifying the *existence* rather than the *magnitude* of influence.

Finally, we do not know how long effects may last into practice. Longitudinal data from a larger sample would be necessary to evaluate this.

## Conclusion

Participation in a single sim-IPE session with an ad hoc interprofessional team has benefits for attitudes towards interprofessional learning, forming a potential virtuous circle of improved engagement.

It can also enhance some aspects of professional identity for participants, notably the positive affect associated with being a student, professional or team member. This may reflect benefits for wellbeing associated with professional identity. Increases in team identity indicate these are ‘real’ groups in psychological terms.

While short-term interprofessional simulation has an educational benefit, more sustained opportunities to work in practice with other professions could lead to greater changes, particularly for medical students.

## Supplementary information

**Additional file 1.** Questionnaire items used in Pre and Post-questionnaires.

**Additional file 2.** Regression analysis R output.

## Data Availability

The datasets supporting the conclusions of this article are available in the Newcastle University data repository doi 10.25405/data.ncl.10029233

## Abbreviations

IPL: Inter-professional learning
RIPLS_Core_: Readiness for inter-professional learning scale _Core_ items
sim-IPE: Simulation-based inter-professional education
Tukey’s HSD: Tukey’s honestly significant difference test
