# Supplementary material for: Effects of a single interprofessional simulation session on medical and nursing students’ attitudes toward interprofessional learning and professional identity: a questionnaire study
Source: BMC Med Educ. 2020 Mar 4;20:65. doi: 10.1186/s12909-020-1971-6 (PMC7057493; doi:10.1186/s12909-020-1971-6)
Supplement: Supplementary file 1 — Additional file 1. Questionnaire items used in Pre and Post-questionnaires. [file 12909_2020_1971_MOESM1_ESM.docx]

**Additional file 1. Questionnaire items used in Pre and Post-questionnaires**

**RIPLS questionnaire (Parsell & Bligh 1999)**

Completed on a 5-point scale (Strongly disagree, Disagree, Undecided, Agree, Strongly Agree) in Study 1 and Study 2.

1. Learning with other students will help me become a more effective member of a health care team
2. Patients would ultimately benefit if health care students worked together to solve patient problems
3. Shared learning with other health care students will increase my ability to understand clinical problems
4. Learning with health care students before qualification would improve relationships after qualification
5. Communication skills should be learned with other health care students
6. Shared learning will help me to think positively about other professionals
7. For small group learning to work, students need to trust and respect each other
8. Team-working skills are essential for all health care students to learn
9. Shared learning will help me to understand my own limitations
10. I don't want to waste my time learning with other health care students
11. It is not necessary for undergraduate health care students to learn together
12. Clinical problem-solving skills can only be learned with students from my own department
13. Shared learning with other health care students will help me to communicate better with patients and other professionals
14. I would welcome the opportunity to work on small-group projects with other health care students
15. Shared learning will help to clarify the nature of patient problems
16. Shared learning before qualification will help me become a better team worker
17. The function of nurses and therapists is mainly to provide support for doctors
18. I'm not sure what my professional role will be
19. I have to acquire much more knowledge and skill than other healthcare students

Items 10, 11, 12 are reverse scored.

Final analysis included only items 3, 4, 8, 11 and 15 (the RIPLS-core suggested by Kerry et al 2018).

**Study 1 Identity Scale: ‘Strength’ (Brown et al 1986)**

Completed on a 7-point scale (Strongly Disagree, Disagree, Disagree somewhat, Neutral, Agree somewhat, Agree, Strongly agree) in Study 1 only.

Considering the group [doctors/nurses], please circle a number to indicate how much you agree with each of these statements

1. I am a person who considers the group important

2. I am a person who identifies with the group

3. I am a person who feels strong ties with the group

4. I am a person who is glad to belong to the group

5. I am a person who sees myself as belonging to the group

6. I am a person who makes excuses for belonging to the group

7. I am a person who tries to hide belonging to the group

8. I am a person who feels held back by the group

9. I am a person who is annoyed to say that I'm a member of the group

10. I am a person who criticizes the group

Items 6-10 are reverse-scored.

**Study 1 Identity Scale: ‘Importance’ (Luhtanen & Crocker 1992)**

Completed on a 7-point scale (Strongly Disagree, Disagree, Disagree somewhat, Neutral, Agree somewhat, Agree, Strongly agree) in Study 1 only

1. Being a [doctor/nurse] is an important part of my self-image

2. Being a [doctor/nurse] is unimportant to my sense of what kind of person I am

3. Being a [doctor/nurse] is an important reflection of who I am

4. Being a [doctor/nurse] has very little to do with how I feel about myself

Items 2 and 4 are reverse-scored.

**Study 2 Identity scale (Cameron 2004)**

Completed on a 5-point scale (Strongly Disagree, Disagree a little, Neither agree nor disagree, Agree a little, Strongly agree) in Study 2 only

Introductory text for each directed to the target:

For the questions on this page, think about the group ‘[doctors/nurses]’, in general.

For the questions on this page, think about the group ‘[medical/nursing students]’, in general.

For the questions on this page, think about the particular interprofessional team you are about to work with/worked with in the simulation scenarios today.

1. I have a lot in common with [target].

2. I feel strong ties to [target].

3. I find it difficult to form a bond with [target].

4. I don’t feel a sense of being “connected” with [target].

5. I often think about the fact that I am [going to be] a [target].

6. Overall, the fact that I am [going to be] a [target] has very little to do with how I feel about myself.

7. In general, the fact that I am [going to be] a [target] is an important part of my self-image.

8. The fact that I am [going to be] a [target] rarely enters my mind.

9. In general, I’m glad I am [going to be] a [target].

10. I often regret that I am [going to be] a [target].

11. I don’t feel good about the fact that I am [going to be] a [target].

12. Generally, I feel good when I think about myself being a [target].

Ingroup Ties subscale – items 1-4

Centrality subscale – items 2-8

Ingroup Affect subscale – items 9-12

Items 3, 4, 6, 8, 10, 11 are reverse-scored.
