# Supplementary material for: Effects of a single interprofessional simulation session on medical and nursing students’ attitudes toward interprofessional learning and professional identity: a questionnaire study
Source: BMC Med Educ. 2020 Mar 4;20:65. doi: 10.1186/s12909-020-1971-6 (PMC7057493; doi:10.1186/s12909-020-1971-6)
Supplement: Supplementary file 2 — Additional file 2. Regression analysis R output. [file 12909_2020_1971_MOESM2_ESM.docx]

**Additional file 2: Regression analysis R output**

Presented below is raw R output for regression and follow up analyses.

Analyses are:

Anova for each model – anova()

Summary table for each model – summary()

Confidence intervals for regression coefficients – confint()

Estimated marginal means for effects of interest – emmeans()

Pairwise comparisons on estimated marginal means for hypothesised effects – pairs()

> library(lme4)

> library(emmeans)

>

**> # FINAL MODELS**

**>**

**> # RIPLS STUDY 1**

>

> ripls.1.final <- lmer(ripls.core ~

+ part.group + pre.post + (1|p.id) +

+ part.group:pre.post,

+ data=iplqstudy1.long, REML = T)

> anova(ripls.1.final)

Analysis of Variance Table

Df Sum Sq Mean Sq F value

part.group 1 2.54650 2.54650 31.659

pre.post 1 2.42246 2.42246 30.117

part.group:pre.post 1 0.70509 0.70509 8.766

> summary(ripls.1.final)

Linear mixed model fit by REML ['lmerMod']

Formula: ripls.core ~ part.group + pre.post + (1 | p.id) + part.group:pre.post

Data: iplqstudy1.long

REML criterion at convergence: 264.6

Scaled residuals:

Min 1Q Median 3Q Max

-3.2760 -0.4459 -0.0047 0.5166 2.8124

Random effects:

Groups Name Variance Std.Dev.

p.id (Intercept) 0.11981 0.3461

Residual 0.08044 0.2836

Number of obs: 250, groups: p.id, 126

Fixed effects:

Estimate Std. Error t value

(Intercept) 4.19826 0.04806 87.361

part.groupNurse 0.32279 0.08706 3.708

pre.postPost 0.12674 0.04315 2.937

part.groupNurse:pre.postPost 0.23116 0.07807 2.961

Correlation of Fixed Effects:

(Intr) prt.gN pr.psP

part.grpNrs -0.552

pre.postPst -0.457 0.252

prt.grpN:.P 0.253 -0.451 -0.553

> confint(ripls.1.final)

2.5 % 97.5 %

.sig01 0.28778636 0.4061960

.sigma 0.24970757 0.3202026

(Intercept) 4.10432740 4.2921606

part.groupNurse 0.15266541 0.4929526

pre.postPost 0.04217174 0.2112496

part.groupNurse:pre.postPost 0.07822345 0.3841450

> ripls.1.emlist <- emmeans(ripls.1.final, ~ part.group:pre.post)

> ripls.1.emlist

part.group pre.post emmean SE df lower.CL upper.CL

Medic Pre 4.20 0.0481 184 4.10 4.29

Nurse Pre 4.52 0.0726 182 4.38 4.66

Medic Post 4.33 0.0477 182 4.23 4.42

Nurse Post 4.88 0.0726 182 4.74 5.02

Degrees-of-freedom method: kenward-roger

Confidence level used: 0.95

> pairs(ripls.1.emlist)[c(1,2,5,6)]

contrast estimate SE df t.ratio p.value

Medic,Pre - Nurse,Pre -0.323 0.0871 183 -3.708 0.0016

Medic,Pre - Medic,Post -0.127 0.0432 123 -2.937 0.0204

Nurse,Pre - Nurse,Post -0.358 0.0651 122 -5.501 <.0001

Medic,Post - Nurse,Post -0.554 0.0869 182 -6.377 <.0001

P value adjustment: tukey method for comparing a family of 4 estimates

**> # RIPLS STUDY 2**

>

> ripls.2.final <- lmer(ripls.core ~

+ pre.post + part.group + site + (1 | p.id) +

+ part.group:site,

+ data= iplqstudy2.long.ripls, REML = T)

> anova(ripls.2.final)

Analysis of Variance Table

Df Sum Sq Mean Sq F value

pre.post 1 4.3341 4.3341 58.540

part.group 1 2.1796 2.1796 29.439

site 1 0.2181 0.2181 2.946

part.group:site 1 0.6501 0.6501 8.781

> summary(ripls.2.final)

Linear mixed model fit by REML ['lmerMod']

Formula: ripls.core ~ pre.post + part.group + site + (1 | p.id) + part.group:site

Data: iplqstudy2.long.ripls

REML criterion at convergence: 353.5

Scaled residuals:

Min 1Q Median 3Q Max

-2.78315 -0.46005 0.00341 0.56787 1.96800

Random effects:

Groups Name Variance Std.Dev.

p.id (Intercept) 0.11613 0.3408

Residual 0.07404 0.2721

Number of obs: 354, groups: p.id, 185

Fixed effects:

Estimate Std. Error t value

(Intercept) 4.07854 0.05744 71.004

pre.postPost 0.22491 0.02935 7.664

part.groupNurse 0.53974 0.10099 5.345

siteOx 0.24298 0.07665 3.170

part.groupNurse:siteOx -0.37147 0.12536 -2.963

Correlation of Fixed Effects:

(Intr) pr.psP prt.gN siteOx

pre.postPst -0.239

part.grpNrs -0.537 0.001

siteOx -0.706 -0.004 0.402

prt.grpNr:O 0.432 0.002 -0.806 -0.611

> confint(ripls.2.final)

2.5 % 97.5 %

.sig01 0.28999893 0.3868579

.sigma 0.24473345 0.3030728

(Intercept) 3.96661887 4.1904611

pre.postPost 0.16728557 0.2826185

part.groupNurse 0.34298046 0.7365732

siteOx 0.09360844 0.3923376

part.groupNurse:siteOx -0.61578084 -0.1272174

> ripls2.prepost.emlist <- emmeans(ripls.2.final, ~ pre.post)

> ripls2.prepost.emlist

pre.post emmean SE df lower.CL upper.CL

Pre 4.38 0.0342 247 4.31 4.44

Post 4.60 0.0350 260 4.53 4.67

Results are averaged over the levels of: part.group, site

Degrees-of-freedom method: kenward-roger

Confidence level used: 0.95

> pairs(ripls2.prepost.emlist)

contrast estimate SE df t.ratio p.value

Pre - Post -0.225 0.0294 173 -7.662 <.0001

Results are averaged over the levels of: part.group, site

> ripls2.site.partgroup.emlist <- emmeans(ripls.2.final, ~ site:part.group)

> ripls2.site.partgroup.emlist

site part.group emmean SE df lower.CL upper.CL

Ncl Medic 4.19 0.0558 180 4.08 4.30

Ox Medic 4.43 0.0526 178 4.33 4.54

Ncl Nurse 4.73 0.0842 181 4.56 4.90

Ox Nurse 4.60 0.0525 183 4.50 4.71

Results are averaged over the levels of: pre.post

Degrees-of-freedom method: kenward-roger

Confidence level used: 0.95

> pairs(ripls2.site.partgroup.emlist)[c(1,2,5,6)]

contrast estimate SE df t.ratio p.value

Ncl,Medic - Ox,Medic -0.243 0.0767 179 -3.170 0.0096

Ncl,Medic - Ncl,Nurse -0.540 0.1010 180 -5.344 <.0001

Ox,Medic - Ox,Nurse -0.168 0.0743 180 -2.265 0.1101

Ncl,Nurse - Ox,Nurse 0.128 0.0992 181 1.295 0.5672

Results are averaged over the levels of: pre.post

P value adjustment: tukey method for comparing a family of 4 estimates

**> ###**

**> ###**

**> ### STUDY 1 IDENTITY - IMPORTANCE**

> importance.final <- lmer(importance ~ part.group + (1 | p.id),

+ data=iplqstudy1.long, REML = T)

> anova(importance.final)

Analysis of Variance Table

Df Sum Sq Mean Sq F value

part.group 1 7.7415 7.7415 26.759

> summary(importance.final)

Linear mixed model fit by REML ['lmerMod']

Formula: importance ~ part.group + (1 | p.id)

Data: iplqstudy1.long

REML criterion at convergence: 624.2

Scaled residuals:

Min 1Q Median 3Q Max

-2.86771 -0.50397 0.03682 0.55927 2.70983

Random effects:

Groups Name Variance Std.Dev.

p.id (Intercept) 0.7200 0.8485

Residual 0.2893 0.5379

Number of obs: 249, groups: p.id, 126

Fixed effects:

Estimate Std. Error t value

(Intercept) 4.56841 0.09928 46.013

part.groupNurse 0.93537 0.18082 5.173

Correlation of Fixed Effects:

(Intr)

part.grpNrs -0.549

> confint(importance.final)

2.5 % 97.5 %

.sig01 0.7212600 0.9798749

.sigma 0.4769095 0.6131177

(Intercept) 4.3738497 4.7629007

part.groupNurse 0.5810863 1.2896397

> importance.emlist <- emmeans(importance.final, ~ part.group)

> importance.emlist

part.group emmean SE df lower.CL upper.CL

Medic 4.57 0.0993 124 4.37 4.76

Nurse 5.50 0.1511 124 5.20 5.80

Degrees-of-freedom method: kenward-roger

Confidence level used: 0.95

> pairs(importance.emlist)

contrast estimate SE df t.ratio p.value

Medic - Nurse -0.935 0.181 124 -5.173 <.0001

**> ### STUDY 1 IDENTITY - STRENGTH**

> strength.final <- lmer(strength ~

+ part.group + pre.post + (1|p.id) +

+ part.group:pre.post ,

+ data=iplqstudy1.long, REML = T)

> anova(strength.final)

Analysis of Variance Table

Df Sum Sq Mean Sq F value

part.group 1 2.10048 2.10048 27.8885

pre.post 1 1.38243 1.38243 18.3548

part.group:pre.post 1 0.35689 0.35689 4.7385

> summary(strength.final)

Linear mixed model fit by REML ['lmerMod']

Formula: strength ~ part.group + pre.post + (1 | p.id) + part.group:pre.post

Data: iplqstudy1.long

REML criterion at convergence: 381.9

Scaled residuals:

Min 1Q Median 3Q Max

-1.94738 -0.46921 0.03916 0.44052 1.89878

Random effects:

Groups Name Variance Std.Dev.

p.id (Intercept) 0.42206 0.6497

Residual 0.07532 0.2744

Number of obs: 244, groups: p.id, 126

Fixed effects:

Estimate Std. Error t value

(Intercept) 5.49928 0.07555 72.790

part.groupNurse 0.61289 0.13784 4.446

pre.postPost 0.10320 0.04227 2.441

part.groupNurse:pre.postPost 0.17110 0.07860 2.177

Correlation of Fixed Effects:

(Intr) prt.gN pr.psP

part.grpNrs -0.548

pre.postPst -0.286 0.157

prt.grpN:.P 0.154 -0.287 -0.538

> confint(strength.final)

2.5 % 97.5 %

.sig01 0.56443937 0.7412221

.sigma 0.24073196 0.3109665

(Intercept) 5.35141993 5.6471209

part.groupNurse 0.34314259 0.8826305

pre.postPost 0.02036486 0.1860052

part.groupNurse:pre.postPost 0.01696294 0.3249597

> strength.prepost.int.emlist <- emmeans(strength.final, ~ pre.post:part.group)

> strength.prepost.int.emlist

pre.post part.group emmean SE df lower.CL upper.CL

Pre Medic 5.50 0.0756 145 5.35 5.65

Post Medic 5.60 0.0753 144 5.45 5.75

Pre Nurse 6.11 0.1153 147 5.88 6.34

Post Nurse 6.39 0.1153 147 6.16 6.61

Degrees-of-freedom method: kenward-roger

Confidence level used: 0.95

> pairs(strength.prepost.int.emlist)[c(1,2,5,6)]

contrast estimate SE df t.ratio p.value

Pre,Medic - Post,Medic -0.103 0.0423 117 -2.441 0.0751

Pre,Medic - Pre,Nurse -0.613 0.1378 146 -4.446 0.0001

Post,Medic - Post,Nurse -0.784 0.1377 146 -5.693 <.0001

Pre,Nurse - Post,Nurse -0.274 0.0663 118 -4.138 0.0004

P value adjustment: tukey method for comparing a family of 4 estimates

**> #### STUDY 2 identity**

**> ####**

**> ##### CENTRALITY ########**

>

> cent.final <- lmer(cent ~

+ pre.post + part.group + site + target + (1 | p.id) +

+ pre.post:target + part.group:site + part.group:target + site:target +

+ part.group:site:target,

+ data=iplqstudy2.long.identity, REML = T)

> anova(cent.final)

Analysis of Variance Table

Df Sum Sq Mean Sq F value

pre.post 1 0.174 0.1738 0.6620

part.group 1 12.388 12.3879 47.1937

site 1 2.138 2.1381 8.1454

target 2 56.167 28.0836 106.9890

pre.post:target 2 3.834 1.9168 7.3025

part.group:site 1 0.505 0.5045 1.9220

part.group:target 2 4.467 2.2337 8.5097

site:target 2 0.097 0.0487 0.1856

part.group:site:target 2 2.449 1.2247 4.6656

> summary(cent.final)

Linear mixed model fit by REML ['lmerMod']

Formula: cent ~ pre.post + part.group + site + target + (1 | p.id) + pre.post:target +

part.group:site + part.group:target + site:target + part.group:site:target

Data: iplqstudy2.long.identity

REML criterion at convergence: 1931.5

Scaled residuals:

Min 1Q Median 3Q Max

-3.6118 -0.5189 0.0583 0.5864 2.7455

Random effects:

Groups Name Variance Std.Dev.

p.id (Intercept) 0.2132 0.4617

Residual 0.2625 0.5123

Number of obs: 1051, groups: p.id, 186

Fixed effects:

Estimate Std. Error t value

(Intercept) 3.540202 0.087471 40.473

pre.postPost 0.003487 0.054174 0.064

part.groupNurse 0.742934 0.149262 4.977

siteOx 0.415792 0.114700 3.625

targetStudent -0.008990 0.082552 -0.109

targetTeam -0.588423 0.082358 -7.145

pre.postPost:targetStudent -0.122270 0.077954 -1.569

pre.postPost:targetTeam 0.180930 0.076706 2.359

part.groupNurse:siteOx -0.441173 0.186149 -2.370

part.groupNurse:targetStudent -0.245330 0.137963 -1.778

part.groupNurse:targetTeam -0.130500 0.133006 -0.981

siteOx:targetStudent -0.090077 0.100645 -0.895

siteOx:targetTeam -0.233175 0.100930 -2.310

part.groupNurse:siteOx:targetStudent 0.158174 0.170982 0.925

part.groupNurse:siteOx:targetTeam 0.496203 0.165398 3.000

> confint(cent.final)

2.5 % 97.5 %

.sig01 0.40303394 0.51758467

.sigma 0.48601433 0.53402237

(Intercept) 3.36995090 3.71044438

pre.postPost -0.10218162 0.10905996

part.groupNurse 0.45240755 1.03351932

siteOx 0.19250907 0.63906549

targetStudent -0.16993219 0.15196075

targetTeam -0.74900561 -0.42786664

pre.postPost:targetStudent -0.27426020 0.02970193

pre.postPost:targetTeam 0.03141346 0.33051477

part.groupNurse:siteOx -0.80354070 -0.07882488

part.groupNurse:targetStudent -0.51448773 0.02347496

part.groupNurse:targetTeam -0.38979593 0.12882344

siteOx:targetStudent -0.28630073 0.10614625

siteOx:targetTeam -0.42994472 -0.03638847

part.groupNurse:siteOx:targetStudent -0.17504724 0.49164653

part.groupNurse:siteOx:targetTeam 0.17373801 0.81866690

> # prepost x target - cent only

> cent.preposttarget.emlist <- emmeans(cent.final, ~ pre.post:target)

> cent.preposttarget.emlist

pre.post target emmean SE df lower.CL upper.CL

Pre Prof 4.01 0.0533 502 3.90 4.11

Post Prof 4.01 0.0544 528 3.91 4.12

Pre Student 3.87 0.0553 548 3.76 3.98

Post Student 3.75 0.0564 576 3.64 3.86

Pre Team 3.36 0.0537 509 3.26 3.47

Post Team 3.55 0.0550 540 3.44 3.66

Results are averaged over the levels of: part.group, site

Degrees-of-freedom method: kenward-roger

Confidence level used: 0.95

> pairs(cent.preposttarget.emlist)[c(1,10,15, 2,4,11, 7,9,14)]

contrast estimate SE df t.ratio p.value

Pre,Prof - Post,Prof -0.00349 0.0542 860 -0.064 1.0000

Pre,Student - Post,Student 0.11878 0.0564 860 2.107 0.4691

Pre,Team - Post,Team -0.18442 0.0547 860 -3.373 0.0220

Pre,Prof - Pre,Student 0.13715 0.0570 863 2.406 0.2818

Pre,Prof - Pre,Team 0.64621 0.0555 858 11.645 <.0001

Pre,Student - Pre,Team 0.50906 0.0572 860 8.902 <.0001

Post,Prof - Post,Student 0.25942 0.0587 860 4.419 0.0004

Post,Prof - Post,Team 0.46528 0.0573 856 8.117 <.0001

Post,Student - Post,Team 0.20586 0.0592 860 3.479 0.0154

Results are averaged over the levels of: part.group, site

P value adjustment: tukey method for comparing a family of 9 estimates

> cent.3way.partgroup.emlist <- emmeans(cent.final, ~ part.group:site:target)

> cent.3way.partgroup.emlist # comparisons

part.group site target emmean SE df lower.CL upper.CL

Medic Ncl Prof 3.54 0.0834 315 3.38 3.71

Nurse Ncl Prof 4.28 0.1238 321 4.04 4.53

Medic Ox Prof 3.96 0.0788 315 3.80 4.11

Nurse Ox Prof 4.26 0.0786 322 4.10 4.41

Medic Ncl Student 3.47 0.0834 315 3.31 3.64

Nurse Ncl Student 3.97 0.1314 380 3.71 4.23

Medic Ox Student 3.80 0.0788 315 3.64 3.95

Nurse Ox Student 4.01 0.0823 374 3.85 4.17

Medic Ncl Team 3.04 0.0836 318 2.88 3.21

Nurse Ncl Team 3.66 0.1258 333 3.41 3.90

Medic Ox Team 3.23 0.0789 317 3.07 3.38

Nurse Ox Team 3.89 0.0789 326 3.74 4.05

Results are averaged over the levels of: pre.post

Degrees-of-freedom method: kenward-roger

Confidence level used: 0.95

> pairs(cent.3way.partgroup.emlist)[c(1, 39, 61, 22, 52,66, 4,8, 42, 15, 19, 49, 25, 29, 55, 34, 38, 60, 2, 13, 40,47, 62, 65)]

contrast estimate SE df t.ratio p.value

**Medic,Ncl,Prof Group - Nurse,Ncl,Prof Group -0.7429 0.1493 319 -4.977 0.0003**

Medic,Ncl,Student Group - Nurse,Ncl,Student Group -0.4976 0.1557 360 -3.197 0.1912

**Medic,Ncl,Team - Nurse,Ncl,Team -0.6124 0.1510 328 -4.056 0.0130**

Medic,Ox,Prof Group - Nurse,Ox,Prof Group -0.3018 0.1112 318 -2.713 0.5087

Medic,Ox,Student Group - Nurse,Ox,Student Group -0.2146 0.1139 344 -1.884 0.9704

**Medic,Ox,Team - Nurse,Ox,Team -0.6675 0.1116 321 -5.982 <.0001**

Medic,Ncl,Prof Group - Medic,Ncl,Student Group. 0.0701 0.0732 854 0.958 1.0000

**Medic,Ncl,Prof Group - Medic,Ncl,Team 0.4980 0.0734 855 6.783 <.0001**

**Medic,Ncl,Student Group - Medic,Ncl,Team 0.4278 0.0734 855 5.827 <.0001**

Nurse,Ncl,Prof Group - Nurse,Ncl,Student Group 0.3155 0.1170 873 2.697 0.5194

**Nurse,Ncl,Prof Group - Nurse,Ncl,Team 0.6285 0.1109 861 5.666 <.0001**

Nurse,Ncl,Student Group - Nurse,Ncl,Team 0.3130 0.1181 869 2.650 0.5569

Medic,Ox,Prof Group - Medic,Ox,Student Group 0.1602 0.0691 854 2.319 0.8029

**Medic,Ox,Prof Group - Medic,Ox,Team 0.7311 0.0693 855 10.554 <.0001**

**Medic,Ox,Student Group - Medic,Ox,Team 0.5709 0.0693 855 8.242 <.0001**

Nurse,Ox,Prof Group - Nurse,Ox,Student Group 0.2474 0.0737 866 3.357 0.1221

**Nurse,Ox,Prof Group - Nurse,Ox,Team 0.3654 0.0698 855 5.236 0.0001**

Nurse,Ox,Student Group - Nurse,Ox,Team 0.1181 0.0741 867 1.593 0.9966

Medic,Ncl,Prof Group - Medic,Ox,Prof Group -0.4158 0.1147 315 -3.625 0.0577

Nurse,Ncl,Prof Group - Nurse,Ox,Prof Group 0.0254 0.1466 321 0.173 1.0000

Medic,Ncl,Student Group - Medic,Ox,Student Group -0.3257 0.1147 315 -2.840 0.4116

Nurse,Ncl,Student Group - Nurse,Ox,Student Group -0.0427 0.1551 379 -0.275 1.0000

Medic,Ncl,Team - Medic,Ox,Team -0.1826 0.1149 317 -1.589 0.9965

Nurse,Ncl,Team - Nurse,Ox,Team -0.2376 0.1484 331 -1.601 0.9961

Results are averaged over the levels of: pre.post

P value adjustment: tukey method for comparing a family of 24 estimates

**> ##### INGROUP TIES ########**

> ties.final <- lmer(ties ~

+ pre.post + part.group + site + target + (1 | p.id) +

+ pre.post:site + pre.post:target + part.group:site +

+ part.group:target + site:target +

+ pre.post:site:target + part.group:site:target,

+ data=iplqstudy2.long.identity, REML = T)

> anova(ties.final)

Analysis of Variance Table

Df Sum Sq Mean Sq F value

pre.post 1 5.7117 5.7117 24.1678

part.group 1 3.1315 3.1315 13.2502

site 1 2.3212 2.3212 9.8218

target 2 23.9693 11.9847 50.7106

pre.post:site 1 1.2960 1.2960 5.4839

pre.post:target 2 12.2941 6.1470 26.0099

part.group:site 1 1.0368 1.0368 4.3871

part.group:target 2 5.1731 2.5866 10.9445

site:target 2 2.3659 1.1830 5.0055

pre.post:site:target 2 1.6550 0.8275 3.5015

part.group:site:target 2 2.0204 1.0102 4.2744

> summary(ties.final)

Linear mixed model fit by REML ['lmerMod']

Formula: ties ~ pre.post + part.group + site + target + (1 | p.id) + pre.post:site +

pre.post:target + part.group:site + part.group:target + site:target +

pre.post:site:target + part.group:site:target

Data: iplqstudy2.long.identity

REML criterion at convergence: 1875.5

Scaled residuals:

Min 1Q Median 3Q Max

-3.7501 -0.5515 0.0703 0.5930 3.3031

Random effects:

Groups Name Variance Std.Dev.

p.id (Intercept) 0.2111 0.4595

Residual 0.2363 0.4861

Number of obs: 1072, groups: p.id, 186

Fixed effects:

Estimate Std. Error t value

(Intercept) 3.49957 0.09077 38.556

pre.postPost 0.10937 0.08191 1.335

part.groupNurse 0.67528 0.14577 4.633

siteOx 0.49066 0.12354 3.972

targetStudent 0.38187 0.08975 4.255

targetTeam 0.03733 0.08957 0.417

pre.postPost:siteOx -0.05366 0.10541 -0.509

pre.postPost:targetStudent -0.26403 0.11608 -2.275

pre.postPost:targetTeam 0.12294 0.11569 1.063

part.groupNurse:siteOx -0.40885 0.18198 -2.247

part.groupNurse:targetStudent -0.21525 0.12681 -1.697

part.groupNurse:targetTeam -0.48805 0.12488 -3.908

siteOx:targetStudent -0.05572 0.12046 -0.463

siteOx:targetTeam -0.52556 0.12029 -4.369

pre.postPost:siteOx:targetStudent 0.19213 0.14914 1.288

pre.postPost:siteOx:targetTeam 0.39671 0.14873 2.667

part.groupNurse:siteOx:targetStudent -0.10384 0.15759 -0.659

part.groupNurse:siteOx:targetTeam 0.33543 0.15596 2.151

> confint(ties.final)

2.5 % 97.5 %

.sig01 0.40259640 0.51397949

.sigma 0.46069049 0.50563178

(Intercept) 3.32302771 3.67610598

pre.postPost -0.05013090 0.26875041

part.groupNurse 0.39163714 0.95894782

siteOx 0.25035578 0.73094529

targetStudent 0.20716311 0.55658355

targetTeam -0.13702412 0.21168585

pre.postPost:siteOx -0.25880353 0.15156702

pre.postPost:targetStudent -0.48999140 -0.03807470

pre.postPost:targetTeam -0.10226543 0.34813666

part.groupNurse:siteOx -0.76293906 -0.05470988

part.groupNurse:targetStudent -0.46208657 0.03160043

part.groupNurse:targetTeam -0.73114049 -0.24495978

siteOx:targetStudent -0.29022188 0.17873780

siteOx:targetTeam -0.75972698 -0.29140894

pre.postPost:siteOx:targetStudent -0.09817137 0.48242809

pre.postPost:siteOx:targetTeam 0.10718434 0.68621536

part.groupNurse:siteOx:targetStudent -0.41057152 0.20292379

part.groupNurse:siteOx:targetTeam 0.03187414 0.63904809

> # pre.post:site:target - ties only

> ties.3way.presitetarget.emlist <- emmeans(ties.final, ~ pre.post:site:target)

> ties.3way.presitetarget.emlist[c(1,2,5,6,9,10, 3,4, 7,8,11,12)]

pre.post site target emmean SE df lower.CL upper.CL

Pre Ncl Prof 3.84 0.0828 481 3.67 4.00

Post Ncl Prof 3.95 0.0844 507 3.78 4.11

Pre Ncl Student 4.11 0.0840 501 3.95 4.28

Post Ncl Student 3.96 0.0852 519 3.79 4.12

Pre Ncl Team 3.63 0.0828 481 3.47 3.79

Post Ncl Team 3.86 0.0847 511 3.70 4.03

Pre Ox Prof 4.12 0.0631 511 4.00 4.25

Post Ox Prof 4.18 0.0645 540 4.05 4.31

Pre Ox Student 4.29 0.0629 507 4.17 4.41

Post Ox Student 4.27 0.0649 549 4.15 4.40

Pre Ox Team 3.56 0.0631 511 3.43 3.68

Post Ox Team 4.13 0.0645 540 4.01 4.26

Results are averaged over the levels of: part.group

Degrees-of-freedom method: kenward-roger

Confidence level used: 0.95

> pairs(ties.3way.presitetarget.emlist)[c(1, 39, 61, 22, 52,66, 4,8, 42, 15, 19, 49, 25, 29, 55, 34, 38, 60)]

contrast estimate SE df z.ratio p.value

Pre,Ncl, Prof Group - Post, Ncl, Prof Group -0.1094 0.0819 Inf -1.335 0.9976

Pre, Ncl,Student Group - Post, Ncl,Student Group 0.1547 0.0828 Inf 1.867 0.9239

Pre, Ncl,Team - Post, Ncl,Team -0.2323 0.0823 Inf -2.824 0.2975

Pre, Ox, Prof Group - Post, Ox,Prof Group -0.0557 0.0664 Inf -0.840 1.0000

Pre, Ox, Prof Group - Post, Ox,Student Group 0.0162 0.0666 Inf 0.243 1.0000

Pre, Ox,Team - Post, Ox,Team -0.5754 0.0664 Inf -8.671 <.0001

Pre, Ncl, Prof Group - Pre, Ncl,Student Group -0.2742 0.0849 Inf -3.230 0.1094

Pre, Ncl, Prof Group - Pre, Ncl,Team 0.2067 0.0837 Inf 2.469 0.5543

Pre, Ncl,Student Group - Pre, Ncl,Team 0.4809 0.0849 Inf 5.664 <.0001

Post, Ncl, Prof Group - Post, Ncl,Student Group -0.0102 0.0870 Inf -0.117 1.0000

Post, Ncl, Prof Group - Post, Ncl,Team 0.0838 0.0865 Inf 0.968 1.0000

Post, Ncl,Student Group - Post, Ncl,Team 0.0940 0.0873 Inf 1.077 0.9998

Pre, Ox, Prof Group - Pre, Ox,Student Group -0.1666 0.0648 Inf -2.569 0.4768

Pre, Ox, Prof Group - Pre, Ox,Team 0.5645 0.0650 Inf 8.684 <.0001

Pre, Ox,Student Group - Pre, Ox,Team 0.7311 0.0648 Inf 11.276 <.0001

Post, Ox, Prof Group - Post, Ox,Student Group -0.0947 0.0675 Inf -1.402 0.9957

Post, Ox, Prof Group - Post, Ox,Team 0.0449 0.0672 Inf 0.669 1.0000

Post, Ox,Student Group - Post, Ox,Team 0.1396 0.0675 Inf 2.067 0.8354

Results are averaged over the levels of: part.group

Degrees-of-freedom method: asymptotic

P value adjustment: tukey method for comparing a family of 18 estimates

> ties.3way.partgroup.emlist <- emmeans(ties.final, ~ part.group:site:target)

> ties.3way.partgroup.emlist

part.group site target emmean SE df lower.CL upper.CL

Medic Ncl Prof 3.55 0.0815 307 3.39 3.71

Nurse Ncl Prof 4.23 0.1209 313 3.99 4.47

Medic Ox Prof 4.02 0.0773 311 3.87 4.17

Nurse Ox Prof 4.28 0.0768 313 4.13 4.44

Medic Ncl Student 3.80 0.0815 307 3.64 3.96

Nurse Ncl Student 4.26 0.1229 331 4.02 4.51

Medic Ox Student 4.31 0.0771 309 4.16 4.46

Nurse Ox Student 4.26 0.0771 318 4.10 4.41

Medic Ncl Team 3.65 0.0817 310 3.49 3.81

Nurse Ncl Team 3.84 0.1209 313 3.60 4.08

Medic Ox Team 3.79 0.0770 307 3.64 3.94

Nurse Ox Team 3.90 0.0771 318 3.75 4.06

Results are averaged over the levels of: pre.post

Degrees-of-freedom method: kenward-roger

Confidence level used: 0.95

> pairs(ties.3way.partgroup.emlist)[c(1, 39, 61, 22, 52,66, 4,8, 42, 15, 19, 49, 25, 29, 55, 34, 38, 60, 2, 13, 40,47, 62, 65)]

contrast estimate SE df t.ratio p.value

**Medic,Ncl,Prof Group - Nurse,Ncl,Prof Group -0.67528 0.1458 311 -4.633 0.0013**

Medic,Ncl,Student Group - Nurse,Ncl,Student Group -0.46003 0.1475 324 -3.119 0.2311

Medic,Ncl,Team - Nurse,Ncl,Team -0.18723 0.1459 312 -1.284 0.9999

Medic,Ox,Prof Group - Nurse,Ox,Prof Group -0.26644 0.1089 312 -2.446 0.7152

Medic,Ox,Student Group - Nurse,Ox,Student Group 0.05265 0.1090 313 0.483 1.0000

Medic,Ox,Team - Nurse,Ox,Team -0.11381 0.1089 312 -1.045 1.0000

Medic,Ncl,Prof Group - Medic,Ncl,Student Group -0.24986 0.0695 872 -3.597 0.0592

Medic,Ncl,Prof Group - Medic,Ncl,Team -0.09880 0.0697 873 -1.418 0.9994

Medic,Ncl,Student Group - Medic,Ncl,Team 0.15106 0.0697 873 2.168 0.8848

Nurse,Ncl,Prof Group - Nurse,Ncl,Student Group -0.03461 0.1061 877 -0.326 1.0000

**Nurse,Ncl,Prof Group - Nurse,Ncl,Team 0.38925 0.1037 872 3.754 0.0351**

**Nurse,Ncl,Student Group - Nurse,Ncl,Team 0.42386 0.1061 877 3.994 0.0148**

**Medic,Ox,Prof Group - Medic,Ox,Student Group -0.29020 0.0661 873 -4.391 0.0030**

Medic,Ox,Prof Group - Medic,Ox,Team 0.22841 0.0659 873 3.466 0.0890

**Medic,Ox,Student Group - Medic,Ox,Team 0.51861 0.0657 872 7.889 <.0001**

Nurse,Ox,Prof Group - Nurse,Ox,Student Group 0.02889 0.0663 873 0.436 1.0000

**Nurse,Ox,Prof Group - Nurse,Ox,Team 0.38103 0.0662 873 5.752 <.0001**

**Nurse,Ox,Student Group - Nurse,Ox,Team 0.35215 0.0666 873 5.286 <.0001**

**Medic,Ncl,Prof Group - Medic,Ox,Prof Group -0.46383 0.1123 309 -4.129 0.0100**

Nurse,Ncl,Prof Group - Nurse,Ox,Prof Group -0.05499 0.1432 313 -0.384 1.0000

**Medic,Ncl,Student Group - Medic,Ox,Student Group -0.50418 0.1122 308 -4.492 0.0024**

Nurse,Ncl,Student Group - Nurse,Ox,Student Group 0.00851 0.1451 328 0.059 1.0000

Medic,Ncl,Team - Medic,Ox,Team -0.13663 0.1123 309 -1.217 0.9999

Nurse,Ncl,Team - Nurse,Ox,Team -0.06321 0.1434 315 -0.441 1.0000

Results are averaged over the levels of: pre.post

P value adjustment: tukey method for comparing a family of 24 estimates

**> ##### INGROUP AFFECT ########**

> affect.final <- lmer(affect ~

+ pre.post + part.group + site + target + (1 | p.id) +

+ part.group:site + part.group:target + site:target +

+ part.group:site:target,

+ data=iplqstudy2.long.identity, REML = T)

> anova(affect.final)

Analysis of Variance Table

Df Sum Sq Mean Sq F value

pre.post 1 1.7252 1.7252 8.9546

part.group 1 11.5194 11.5194 59.7914

site 1 2.4808 2.4808 12.8767

target 2 0.8264 0.4132 2.1448

part.group:site 1 1.3002 1.3002 6.7489

part.group:target 2 3.9258 1.9629 10.1883

site:target 2 2.9039 1.4519 7.5363

part.group:site:target 2 1.8927 0.9464 4.9121

> summary(affect.final)

Linear mixed model fit by REML ['lmerMod']

Formula: affect ~ pre.post + part.group + site + target + (1 | p.id) +

part.group:site + part.group:target + site:target + part.group:site:target

Data: iplqstudy2.long.identity

REML criterion at convergence: 1669.2

Scaled residuals:

Min 1Q Median 3Q Max

-4.5255 -0.4283 0.1126 0.4972 4.8493

Random effects:

Groups Name Variance Std.Dev.

p.id (Intercept) 0.1928 0.4390

Residual 0.1927 0.4389

Number of obs: 1074, groups: p.id, 186

Fixed effects:

Estimate Std. Error t value

(Intercept) 3.75602 0.07759 48.406

pre.postPost 0.08316 0.02712 3.066

part.groupNurse 0.89667 0.13659 6.565

siteOx 0.48401 0.10523 4.599

targetStudent -0.09689 0.06289 -1.541

targetTeam 0.28012 0.06308 4.441

part.groupNurse:siteOx -0.47629 0.17042 -2.795

part.groupNurse:targetStudent 0.01032 0.11461 0.090

part.groupNurse:targetTeam -0.45626 0.11286 -4.043

siteOx:targetStudent 0.09363 0.08647 1.083

siteOx:targetTeam -0.31748 0.08661 -3.666

part.groupNurse:siteOx:targetStudent -0.07987 0.14223 -0.562

part.groupNurse:siteOx:targetTeam 0.33808 0.14076 2.402

> confint(affect.final)

2.5 % 97.5 %

.sig01 0.38541164 0.49008014

.sigma 0.41716007 0.45781862

(Intercept) 3.60491426 3.90712256

pre.postPost 0.03020871 0.13610399

part.groupNurse 0.63067714 1.16267874

siteOx 0.27908818 0.68894674

targetStudent -0.21970899 0.02584163

targetTeam 0.15694917 0.40323363

part.groupNurse:siteOx -0.80819461 -0.14442536

part.groupNurse:targetStudent -0.21339882 0.23404611

part.groupNurse:targetTeam -0.67653685 -0.23591865

siteOx:targetStudent -0.07513657 0.26246505

siteOx:targetTeam -0.48652025 -0.14838324

part.groupNurse:siteOx:targetStudent -0.35752639 0.19777891

part.groupNurse:siteOx:targetTeam 0.06327007 0.61281601

> # prepost - affect only

> affect.prepost.emlist <- emmeans(affect.final, ~ pre.post)

> affect.prepost.emlist

pre.post emmean SE df lower.CL upper.CL

Pre 4.30 0.0396 227 4.22 4.38

Post 4.38 0.0401 238 4.30 4.46

Results are averaged over the levels of: part.group, site, target

Degrees-of-freedom method: kenward-roger

Confidence level used: 0.95

> pairs(affect.prepost.emlist)

contrast estimate SE df t.ratio p.value

Pre - Post -0.0832 0.0271 891 -3.066 0.0022

Results are averaged over the levels of: part.group, site, target

> affect.3way.partgroup.emlist <- emmeans(affect.final, ~ part.group:site:target)

> affect.3way.partgroup.emlist

part.group site target emmean SE df lower.CL upper.CL

Medic Ncl Prof 3.80 0.0765 298 3.65 3.95

Nurse Ncl Prof 4.69 0.1132 301 4.47 4.92

Medic Ox Prof 4.28 0.0722 297 4.14 4.42

Nurse Ox Prof 4.70 0.0719 301 4.56 4.84

Medic Ncl Student 3.70 0.0764 295 3.55 3.85

Nurse Ncl Student 4.61 0.1150 317 4.38 4.83

Medic Ox Student 4.28 0.0721 295 4.14 4.42

Nurse Ox Student 4.63 0.0722 305 4.49 4.77

Medic Ncl Team 4.08 0.0765 298 3.93 4.23

Nurse Ncl Team 4.52 0.1132 301 4.30 4.74

Medic Ox Team 4.24 0.0721 295 4.10 4.39

Nurse Ox Team 4.55 0.0720 303 4.40 4.69

Results are averaged over the levels of: pre.post

Degrees-of-freedom method: kenward-roger

Confidence level used: 0.95

> pairs(affect.3way.partgroup.emlist)[c(1, 39, 61, 22, 52,66, 4,8, 42, 15, 19, 49, 25, 29, 55, 34, 38, 60, 2, 13, 40,47, 62, 65)]

contrast estimate SE df t.ratio p.value

**Medic,Ncl,Prof Group - Nurse,Ncl,Prof Group -0.89667 0.1366 300 -6.564 <.0001**

**Medic,Ncl,Student Group - Nurse,Ncl,Student Group -0.90699 0.1380 310 -6.572 <.0001**

Medic,Ncl,Team - Nurse,Ncl,Team -0.44041 0.1366 300 -3.224 0.1799

**Medic,Ox,Prof Group - Nurse,Ox,Prof Group -0.42038 0.1019 299 -4.125 0.0103**

Medic,Ox,Student Group - Nurse,Ox,Student Group -0.35083 0.1020 300 -3.439 0.1012

Medic,Ox,Team - Nurse,Ox,Team -0.30219 0.1019 299 -2.965 0.3240

Medic,Ncl,Prof Group - Medic,Ncl,Student Group 0.09689 0.0629 879 1.541 0.9979

**Medic,Ncl,Prof Group - Medic,Ncl,Team -0.28012 0.0631 880 -4.441 0.0024**

**Medic,Ncl,Student Group - Medic,Ncl,Team -0.37701 0.0629 879 -5.995 <.0001**

Nurse,Ncl,Prof Group - Nurse,Ncl,Student Group 0.08657 0.0958 884 0.904 1.0000

Nurse,Ncl,Prof Group - Nurse,Ncl,Team 0.17614 0.0936 879 1.882 0.9719

Nurse,Ncl,Student Group - Nurse,Ncl,Team 0.08957 0.0958 884 0.935 1.0000

Medic,Ox,Prof Group - Medic,Ox,Student Group 0.00326 0.0593 879 0.055 1.0000

Medic,Ox,Prof Group - Medic,Ox,Team 0.03735 0.0593 879 0.629 1.0000

Medic,Ox,Student Group - Medic,Ox,Team 0.03409 0.0592 879 0.576 1.0000

Nurse,Ox,Prof Group - Nurse,Ox,Student Group 0.07281 0.0598 880 1.218 1.0000

Nurse,Ox,Prof Group - Nurse,Ox,Team 0.15554 0.0596 879 2.609 0.5901

Nurse,Ox,Student Group - Nurse,Ox,Team 0.08272 0.0599 880 1.380 0.9996

**Medic,Ncl,Prof Group - Medic,Ox,Prof Group -0.48401 0.1052 297 -4.599 0.0015**

Nurse,Ncl,Prof Group - Nurse,Ox,Prof Group -0.00772 0.1341 301 -0.058 1.0000

**Medic,Ncl,Student Group - Medic,Ox,Student Group -0.57764 0.1050 295 -5.500 <.0001**

Nurse,Ncl,Student Group - Nurse,Ox,Student Group -0.02147 0.1357 314 -0.158 1.0000

Medic,Ncl,Team - Medic,Ox,Team -0.16653 0.1051 296 -1.584 0.9966

Nurse,Ncl,Team - Nurse,Ox,Team -0.02832 0.1341 301 -0.211 1.0000

Results are averaged over the levels of: pre.post

P value adjustment: tukey method for comparing a family of 24 estimates
